# Supplementary material for: Biosynthesis of Amino Acids in Xanthomonas oryzae pv. oryzae Is Essential to Its Pathogenicity
Source: Microorganisms. 2019 Dec 13;7(12):693. doi: 10.3390/microorganisms7120693 (PMC6956189; doi:10.3390/microorganisms7120693)
Supplement: Supplementary file 1 [file microorganisms-07-00693-s001.zip › Fig S1N.docx]

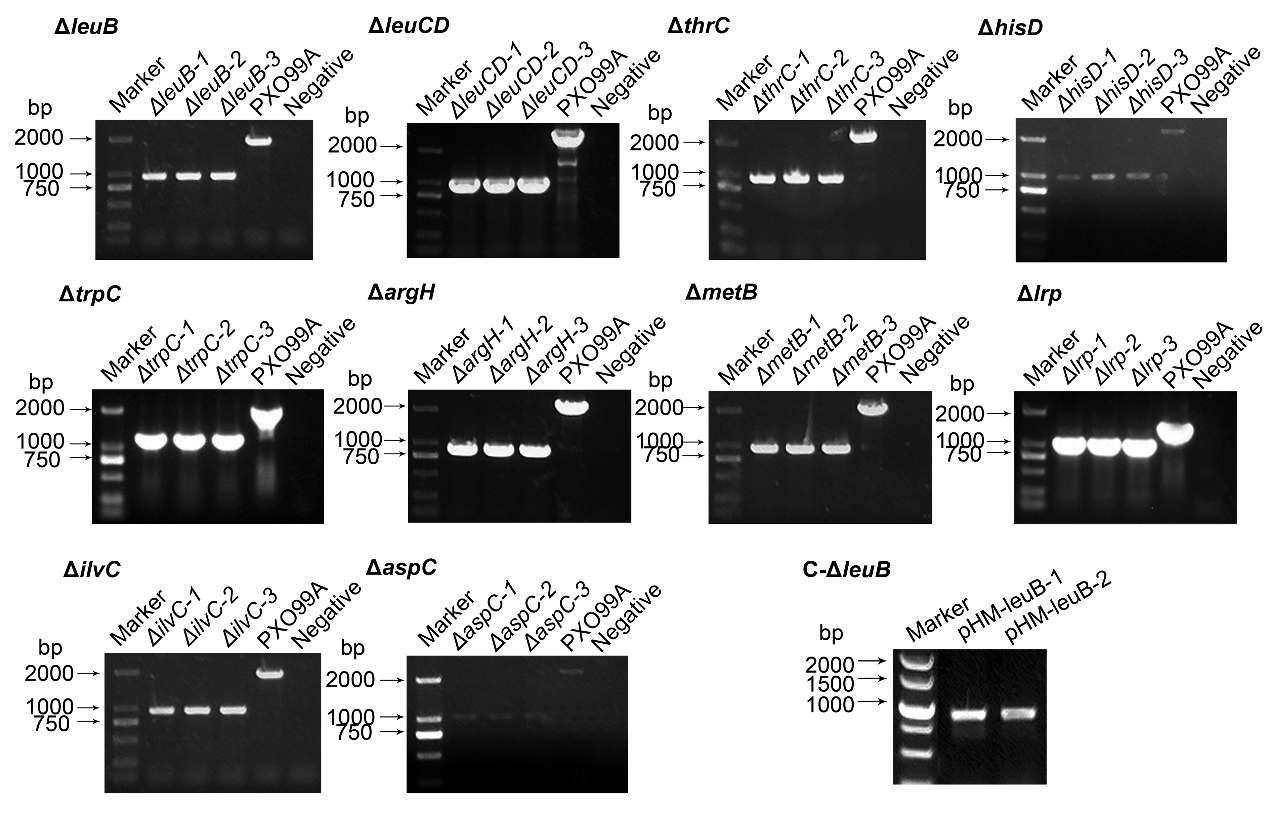


**Figure S1. PCR conformation of in-frame deletion mutants and Δ*leuB* complementary strains.**

For mutants, the primer pairs located ~500 bp up- and down-stream of target genes were used and the PCR fragments of mutants would be shorter than those of the wild type strain PXO99A. For complementary strains, the primer pairs located at the translational start and end sites of *leuB* were used and about 900 bp PCR products were obtained.
