# Supplementary material for: Biosynthesis of Amino Acids in Xanthomonas oryzae pv. oryzae Is Essential to Its Pathogenicity
Source: Microorganisms. 2019 Dec 13;7(12):693. doi: 10.3390/microorganisms7120693 (PMC6956189; doi:10.3390/microorganisms7120693)
Supplement: Supplementary file 1 [file microorganisms-07-00693-s001.zip › Fig S2N.docx]

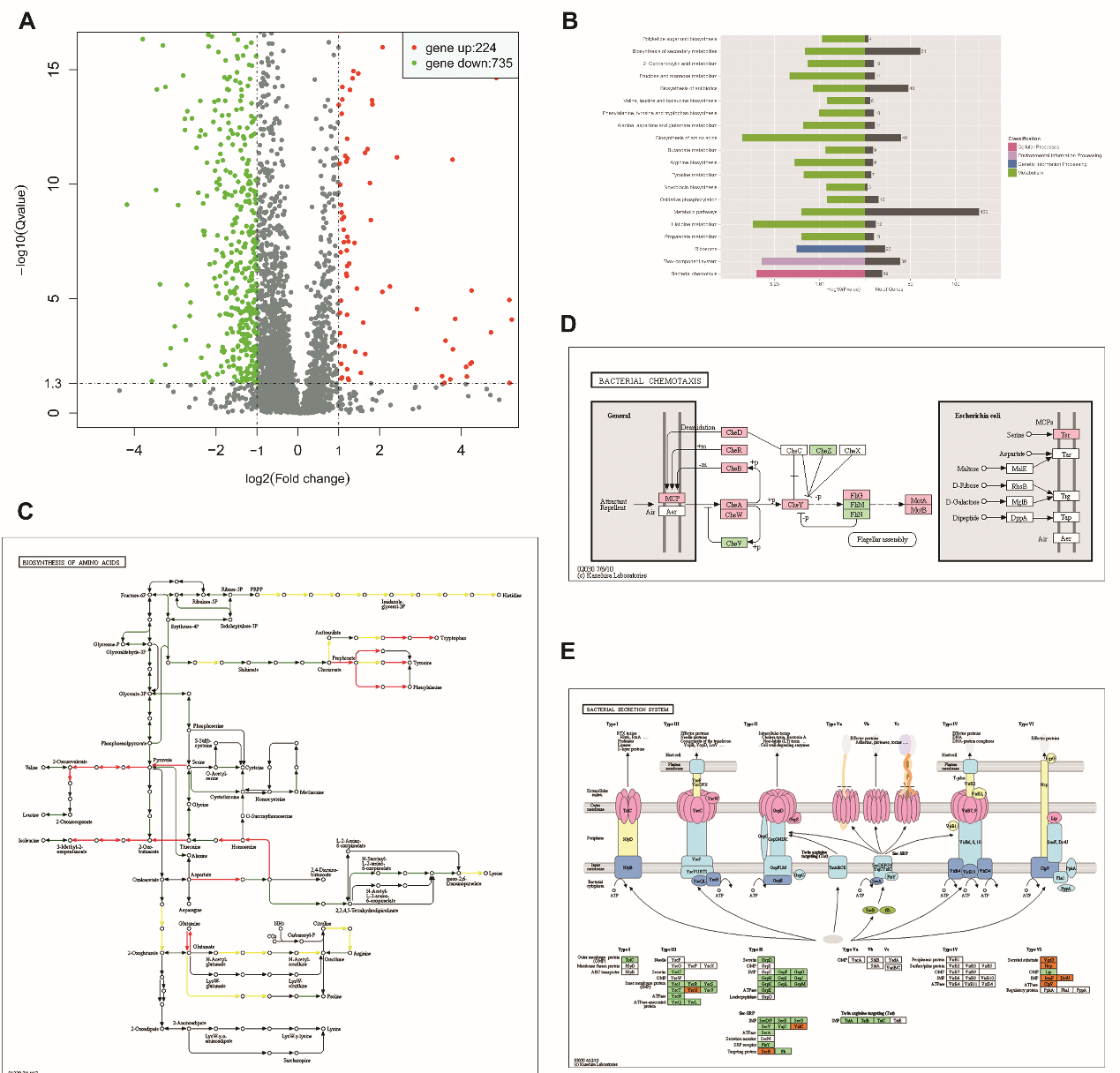


**Figure S2. RNA-Seq indicates the KEGG pathways regulated by leucine.**

(A) The regulted gene numbers by lack of leucine. (B) KEGG pathways regulated by lack of leucine. (C) Amino acid synthesis pathway regulated by leucine. (D) Chemotaxis pathway regulated by leucine. (E) Secrection system pathway regulated by leucine.
