# Supplementary material for: Biosynthesis of Amino Acids in Xanthomonas oryzae pv. oryzae Is Essential to Its Pathogenicity
Source: Microorganisms. 2019 Dec 13;7(12):693. doi: 10.3390/microorganisms7120693 (PMC6956189; doi:10.3390/microorganisms7120693)
Supplement: Supplementary file 1 [file microorganisms-07-00693-s001.zip › Fig S3N.docx]

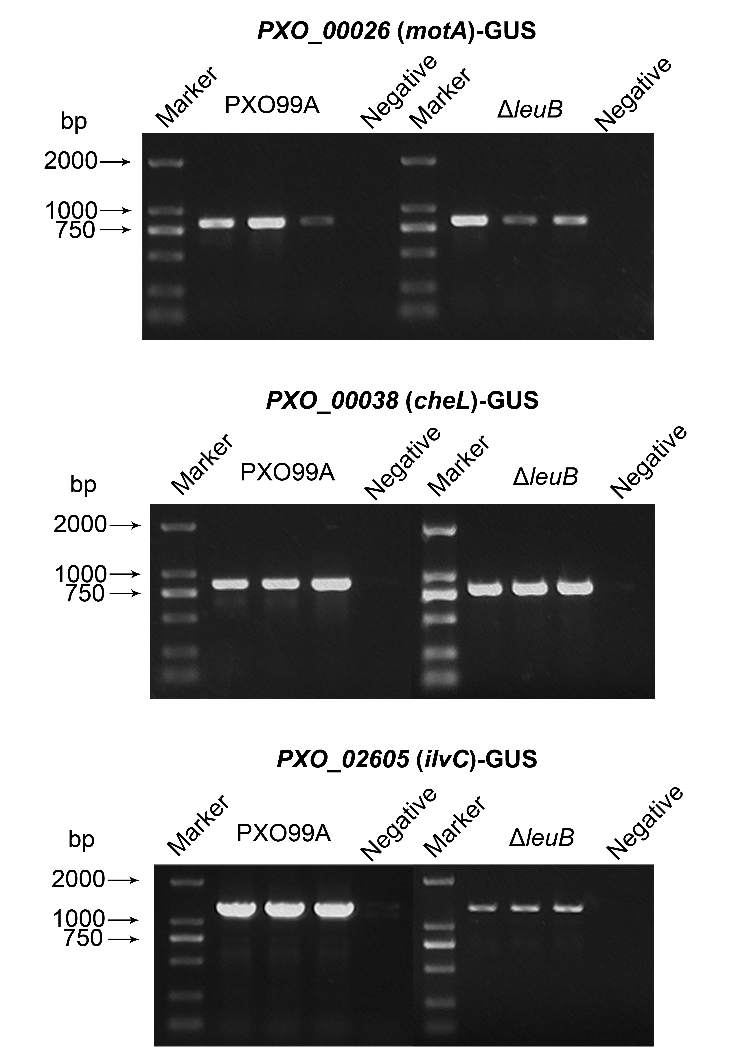


**Figure S3. Construction of the promoter*-gusA* fusion strains.**

Primer pairs including the promoter upstream primer and gusAseqR (located at about 200 bp downstream of *gusA* translational initiational site) were used. Three biological replicates were used.
