# Supplementary material for: Biosynthesis of Amino Acids in Xanthomonas oryzae pv. oryzae Is Essential to Its Pathogenicity
Source: Microorganisms. 2019 Dec 13;7(12):693. doi: 10.3390/microorganisms7120693 (PMC6956189; doi:10.3390/microorganisms7120693)
Supplement: Supplementary file 1 [file microorganisms-07-00693-s001.zip › table S1.doc]

**Table S1. Bacterial strains and plasmids used in this study**

| Name | Relevant genotype or description | Reference |
| --- | --- | --- |
| Strains |  |  |
| ***Xoo*** |  |  |
| PXO99A | Philippine race 6 (PR6) strain, avirulent on Xa21 rice plants, wild type |  |
| Δ*leuB* | Full length deletion of *leuB* (*PXO_02610*) | This study |
| Δ*leuCD* | Full length deletion of *leuC* (*PXO_02613*) and *leuD* (*PXO_02612*) | This study |
| Δ*thrC* | Full length deletion of *thrC* (*PXO_00822*) | This study |
| Δ*hisD* | Full length deletion of *hisD* (*PXO_00833*) | This study |
| Δ*trpC* | Full length deletion of *trpC* (*PXO_04004*) | This study |
| Δ*argH* | Full length deletion of *argH* (*PXO_00346*) | This study |
| Δ*metB* | Full length deletion of *metB* (*PXO_03157*) | This study |
| Δ*lrp* | Full length deletion of *lrp* (*PXO_03072*) | This study |
| Δ*ilvC* | Full length deletion of *ilvC* (*PXO_02605*) | This study |
| Δ*aspC* | Full length deletion of *aspC* (*PXO_02420*) | This study |
| ***E. coli*** |  |  |
| DH5α | φ80d*lacZ*∆M15, ∆(*lacZYA-argF*)U169, *deoR*, *recA1*, *endA1*, *hsdR17*(rk-,mk+), *phoA*, *supE44*, λ-, *thi-1*, *gyrA96* | Lab collection |
| **Plasmids** |  |  |
| pK18*mobsacB* | Suicide plasmid in *Xanthomonas*, Km^R^ |  |
| pHM1 | broad-host range vector, Sp^R^ | Huynh et al., 1989 |
| pK18-*leuB* | pK18*mobsacB* based plasmid for *leuB* deletion | This study |
| pK18-*leuCD* | pK18*mobsacB* based plasmid for *leuCD* deletion | This study |
| pK18-*thrC* | pK18*mobsacB* based plasmid for *thrC* deletion | This study |
| pK18-*hisD* | pK18*mobsacB* based plasmid for *hisD* deletion | This study |
| pK18-*trpC* | pK18*mobsacB* based plasmid for *trpC* deletion | This study |
| pK18-*argH* | pK18*mobsacB* based plasmid for argH deletion | This study |
| pK18-*metB* | pK18*mobsacB* based plasmid for metB deletion | This study |
| pK18-*lrp* | pK18*mobsacB* based plasmid for lrp deletion | This study |
| pK18-*ilvC* | pK18*mobsacB* based plasmid for ilvC deletion | This study |
| pK18-*aspC* | pK18*mobsacB* based plasmid for aspC deletion | This study |
| pHM-*leuB* | Complementary vector containing full length leuB gene, SpR | This study |
| PX0026gus | PX0026-*gusA* | This study |
| PX0038gus | PX0038-*gusA* | This study |
| PX2605gus | PX2605-*gusA* | This study |

Km^R^ and Sp^R^ indicate resistance to Kanamycin and Spectinomycin, respectively

**References**

Hopkins, C.M., White, F.F., Choi, S.H., Guo, A., and Leach, J.E. 1992. Identification of a family of avirulence genes from Xanthomonas oryzae pv. oryzae. Mol Plant Microbe Interact 5:451-459.

Schafer, A., Tauch, A., Jager, W., Kalinowski, J., Thierbach, G., and Puhler, A. 1994. Small mobilizable multi-purpose cloning vectors derived from the Escherichia coli plasmids pK18 and pK19: selection of defined deletions in the chromosome of Corynebacterium glutamicum. Gene 145:69-73.

Huynh, T. V., Dahlbeck, D., & Staskawicz, B. J. 1989. Bacterial blight of soybean: regulation of a pathogen gene determining host cultivar specificity. Science. 245(4924): 1374-1377.
