# Supplementary material for: Biosynthesis of Amino Acids in Xanthomonas oryzae pv. oryzae Is Essential to Its Pathogenicity
Source: Microorganisms. 2019 Dec 13;7(12):693. doi: 10.3390/microorganisms7120693 (PMC6956189; doi:10.3390/microorganisms7120693)
Supplement: Supplementary file 1 [file microorganisms-07-00693-s001.zip › table S2.doc]

**Table S2. Oligonucleotide primers used in this study**

| name | sequence | Application for Amplification |
| --- | --- | --- |
| For deletion | | |
| PX2610ddFF | CATGAATTCCACGCACCTGCAGGCAATCG | Upstream of *leuB* |
| PX2610ddFR | CATTCTAGACAGCTCGTCGTACACCAGCTC |  |
| PX2610ddRF | GGTTCTAGAGCAGTCGGCAAGGTGCTCGAC | Downstream of *leuB* |
| PX2610ddRR | CGAAAGCTTCTGGTCATTCGCTACACGATG |  |
| PX0346ddFF | GCTCAAGCTTATGCACGAGCGCGAGGTATC | Upstream of *argH* |
| PX0346ddFR | AGTCCTCGAGGTCTCGAGTAAGTCGTCCAG |  |
| PX0346ddRF | AGTCCTCGAGACCTGCAACTGGACGTGTTG | Downstream of *argH* |
| PX0346ddRR | GACCGAATTCAGGTGTGCGTTCGTTACGAC |  |
| PX0822ddFF | GCTCAAGCTTACTTCGCTGATCTGCATGAC | Upstream of *thrC* |
| PX0822ddFR | AGTCCTCGAGGATGAACATCAGTGTGACTC |  |
| PX0822ddRF | AGTCCTCGAGCTGCTTGCGGATGCGACCTG | Downstream of *thrC* |
| PX0822ddRR | GACCGAATTCGCTGCTTGTCCAGATCGAAC |  |
| PX0833ddFF | GCTCAAGCTTGGCTGGAGCGTCAAGGCATC | Upstream of *hisD* |
| PX0833ddFR | AGTCCTCGAGAGAATCTTCATGCCAGCGAG |  |
| PX0833ddRF | AGTCCTCGAGCATGCCAATGCAGTAGCACT | Downstream of *hisD* |
| PX0833ddRR | GACCGAATTCGGATATCCGCATGGAAACCA |  |
| PX4004ddFF | GCACAAGCTTGACGGCATGGACGAACTCTC | Upstream of *trpC* |
| PX4004ddFR | GCACGGATCCCAAGAATGGTGTTGAGGATG |  |
| PX4004ddRF | GCACGGATCCTGGTTGGCGAGACCTTCATG | Downstream of *trpC* |
| PX4004ddRR | GGACGAATTCTCTTCGTTATGGCAGTGACG |  |
| PX3157ddFF | GCTCAAGCTTCTGCGCGACCTGATCCTTC | Upstream of *metB* |
| PX3157ddFR | AGTCCTCGAGGATCGCCGTCATGGCTATGA |  |
| PX3157ddRF | AGTCCTCGAGGATTTGCGTGGGGATTTGGA | Downstream of *metB* |
| PX3157ddRR | GACCGAATTCGTCCAAGCATTGAGCGCAAG |  |
| PX3072ddFF | GCACAAGCTTAGTCGCGACTGTAGTCGGAC | Upstream of *lrp* |
| PX3072ddFR | GAACGGATCCATGGAATTTGTCTTGGCAAG |  |
| PX3072ddRF | GAACGGATCCTTGCCGATCGCCGATTGAGG | Downstream of *lrp* |
| PX3072ddRR | GCACGAATTCGTCATGGCGATCCATCCATC |  |
| PX2605ddFF | GTACAAGCTTACATCGTGGTTACTGCGATG | Upstream of *ilvC* |
| PX2605ddFR | CAACGGATCCTTGGGTGTCGTTGCTCATGA |  |
| PX2605ddRF | CAACGGATCCCTCAACGGCGAACAGCAAGC | Downstream of *ilvC* |
| PX2605ddRR | CAGCGAATTCGAACACGTCCAGTTCCTGGA |  |
| PX2420ddFF | GCTCAAGCTTATCACCTGCTGGTGGTAATG | Upstream of *aspC* |
| PX2420ddFR | AGTCCTCGAGGCTTTTGCGGGGCAGTGGAC |  |
| PX2420ddRF | AGTCCTCGAGCAAGGTGGTTCCACTCAAGT | Downstream of *aspC* |
| PX2420ddRR | GACCGAATTCTCGCGTTGAAGGCATCGATC |  |
| PX2612ddFF | ACCTGAATTCCAGCAGCGCGATGCGTTTGC | Upstream of *leuCD* |
| PX2612ddFR | ACCTGGATCCTCGTCGGAAATCGACTCCAG |  |
| PX2613ddRF | ACCTGGATCCCAAGCACTGCCTGCTCAACG | Downstream of *leuCD* |
| PX2613ddRR | ACCTAAGCTTGACTGACCACTGCATCCATG |  |
| For promoter-gus fusion and complementary assays | | |
| PX0026gusF | GTACAAGCTTGACCTGAAATGATCAACACG | 5΄primer of *PXO_00026* (*motA*) promoter |
| PX0026gusR | CAACGGATCCCACGATCGCCAGCACCAGTC | 3΄primer of *PXO_00026* (*motA*) promoter |
| PX0038gusF | GTACAAGCTTAGCCACGCCGTCAGA | 5΄primer of *PXO_00038* (*cheL*) promoter |
| PX0038gusR | CAACGGATCCACAGCTGGCGATCACAGATG | 3΄primer of *PXO_00038* (*cheL*) promoter |
| PX2605gusF | GTACAAGCTTCGACAGCGACATATGTCC | 5΄primer of *PXO_02605* (*ilvC*) promoter |
| PX2605gusR | CAACGGATCCCGGTTGGGTGTCGTTGCTCA | 3΄primer of *PXO_02605* (*ilvC*) promoter |
| PX2610hmF | AACAGCTATGACCATGATTACGCCAAGCTTGAGCAAGCAGATTCTGATTCT | Primers for Δ*leuB* complement |
| PX2610hmR | CGACGTTGTAAAACGACGGCCAGTGAATTCAAGCTTGAAGCGAGCGCTTTTACAGC |  |
| For qPCR analysis | | |
| PX0256qF | AGCGGAGAACGACCATTTCG | qPCR primers for *PXO_00256* (*vgrG*) |
| PX0256qR | CCTGGATGCGGAGTGTCTTG |  |
| PX0264qF | AACTCAGCGAAGGCGTGAT | qPCR primers for *PXO_00264* (*hcp*) |
| PX0264qR | TTCTTGCCCTTGTCGTCCTG |  |
| PX0029qF | TCAAGGTGCAGGAAGTGGTC | qPCR primers for *PXO_00029* (*cheW*) |
| PX0029qR | ATATCTACCGGCGAAGAGCC |  |
| PX0055qF | CCTGACCTCGTTCTTCCGTG | qPCR primers for *PXO_00055* (*cheR*) |
| PX0055qR | CCGTGATGGCGATGGAGTAG |  |
| PX1007qF | AGTCCAAGGGCGAAAACCTG | qPCR primers for *PXO_01007* (*flgG*) |
| PX1007qR | ATTGACGTTGCTGCCTTCCA |  |
| PX6210qF | CCCGTTCGATTTCGTGGTCA | qPCR primers for *PXO_06210* (*cheY*) |
| PX6210qR | ATGATCTGCTCGCGCTTGG |  |
| PX3941qF | GATGCGCTGGTGTGCATTTC | qPCR primers for *PXO_03941* (*ilvD*) |
| PX3941qR | TCAGGTCGAGTTTGTGCTCG |  |
| PX2605qF | CGGCACCTTCACCAAGAACT | qPCR primers for *PXO_02605* (*ilvC*) |
| PX2605qR | CCGTTGAGCCACACCATCTT |  |
| PX0835qF | GGCCGTATTTCGTGTTCGAC | qPCR primers for *PXO_00835* (*hisB*) |
| PX0835qR | CACCGTCAGATGCAGGTTCA |  |
| PX0728qF | GCCCGACTGGTTAGAACTCC | qPCR primers for *PXO_00728* (*hutG*) |
| PX0728qR | GAGCTGATGCACCCACCAAT |  |
| PX3319qF | GGCATCAGCTACAAGGGCAT | qPCR primers for *PXO_03319* (*gltB*) |
| PX3319qR | CGTGTTGGTGGAAAAACGCT |  |
| PX1963qF | GGGTTCATCCAGTCACGTCC | qPCR primers for *PXO_01963* (*gluL*) |
| PX1963qR | TATCGCCCGCGTATTTGAGG |  |
| XoRpoDqF | GGCCAACGAATACGCCAAGT | qPCR primers for *rpoD* (reference gene) |
| XoRpoDqR | TGAGCGTGGTGAAGATGGC |  |

*All primers were designed based on *Xoo* strain PXO99^A^; Restriction enzyme sites are underlined.
